# 8-Meth­oxy-2*H*-chromene-3-carbaldehyde

**DOI:** 10.1107/S1600536812047319

**Published:** 2012-11-24

**Authors:** Dongsoo Koh

**Affiliations:** a Department of Applied Chemistry, Dongduk Women’s University, Seoul 136-714, Republic of Korea

## Abstract

In the title mol­ecule, C_11_H_10_O_3_, the fused dihydro­pyran ring is in a half-chair conformation with the O atom and the methyl­ene C atom positioned 0.1318 (13) and 0.143 (2) Å, respectively, on either side of the mean plane formed by the other four atoms. In the crystal, weak C—H⋯O hydrogen bonds link mol­ecules along [001].

## Related literature

For the synthesis and biological properties of chromene derivatives, see: Mun *et al.* (2012 ▶); Kallikat *et al.* (2011 ▶); Zhang *et al.* (2009 ▶); Gebhardt *et al.* (2007 ▶); Yoon *et al.* (2012 ▶). For the chromene group in natural products, see: Escandón-Rivera *et al.* (2012 ▶); Chen *et al.* (2008 ▶). For related structures, see: Yusufzai *et al.* (2012 ▶); Betz *et al.* (2011 ▶); Bardajee *et al.* (2007 ▶).
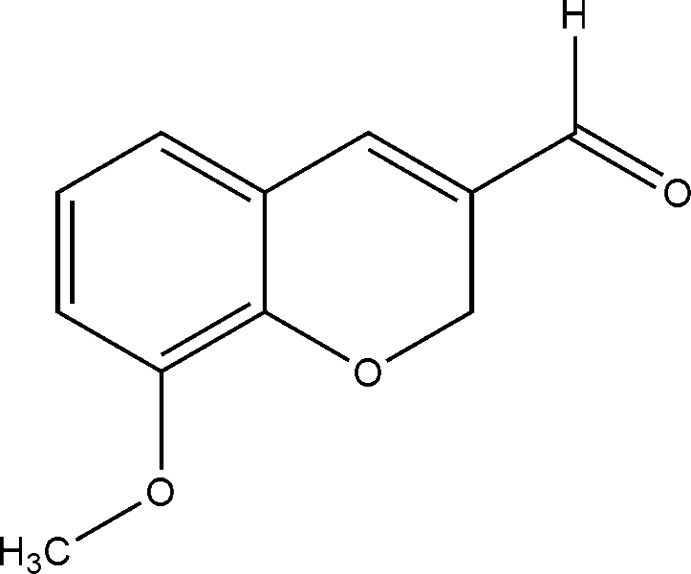


## Experimental

### 

#### Crystal data

C_11_H_10_O_3_
*M* *_r_* = 190.19
Orthorhombic, 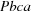

*a* = 6.8940 (6) Å
*b* = 13.2079 (11) Å
*c* = 20.0964 (16) Å
*V* = 1829.9 (3) Å^3^
*Z* = 8
Mo *K*α radiation
μ = 0.10 mm^−1^
*T* = 200 K
0.23 × 0.21 × 0.19 mm

#### Data collection

Bruker SMART CCD diffractometer
12690 measured reflections
2276 independent reflections
1194 reflections with *I* > 2σ(*I*)
*R*_int_ = 0.056

#### Refinement

*R*[*F* ^2^ > 2σ(*F* ^2^)] = 0.051
*wR*(*F* ^2^) = 0.158
*S* = 0.92
2276 reflections
128 parameters
H-atom parameters constrained
Δρ_max_ = 0.28 e Å^−3^
Δρ_min_ = −0.28 e Å^−3^

### 

Data collection: *SMART* (Bruker, 2000 ▶); cell refinement: *SAINT* (Bruker, 2000 ▶); data reduction: *SAINT*; program(s) used to solve structure: *SHELXS97* (Sheldrick, 2008 ▶); program(s) used to refine structure: *SHELXL97* (Sheldrick, 2008 ▶); molecular graphics: *PLATON* (Spek, 2009 ▶); software used to prepare material for publication: *SHELXTL* (Sheldrick, 2008 ▶).

## Supplementary Material

Crystal structure: contains datablock(s) I, global. DOI: 10.1107/S1600536812047319/lh5559sup1.cif

Structure factors: contains datablock(s) I. DOI: 10.1107/S1600536812047319/lh5559Isup2.hkl

Supplementary material file. DOI: 10.1107/S1600536812047319/lh5559Isup3.cml

Additional supplementary materials:  crystallographic information; 3D view; checkCIF report

## Figures and Tables

**Table 1:** Hydrogen-bond geometry (Å, °)

*D*—H⋯*A*	*D*—H	H⋯*A*	*D*⋯*A*	*D*—H⋯*A*
C6—H6*B*⋯O1^i^	0.98	2.49	3.340 (3)	145

Symmetry code: (i) 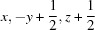.

**Fig. 1.**
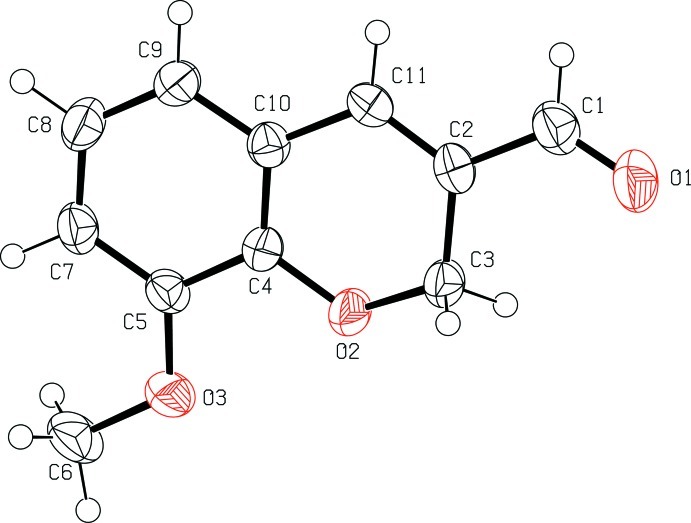
The molecular structure of the title compound, showing displacement ellipsoids drawn at the 50% probability level.

**Fig. 2.**
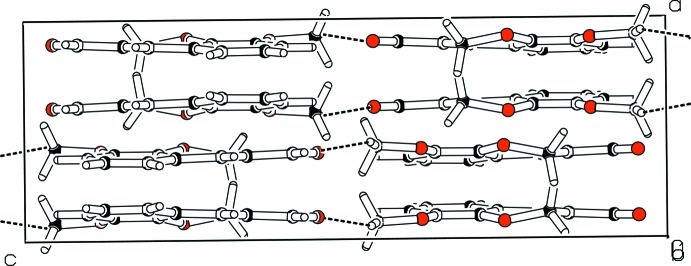
Part of the crystal structure with weak intermolecular C—H···O hydrogen bonds shown as dashed lines.

## supplementary crystallographic information

### Comment

Chromenes have been important heterocyclic components in biologically active pharmaceuticals which show anti-inflammatory (Gebhardt *et al.* 2007) and anticancer (Mun *et al.*, 2012) activities. The 2*H*-chromene skeleton is a core structure of oxygen heterocycles in many natural products having biological activities (Escandón-Rivera *et al.*, 2012; Chen *et al.*, 2008). In a continuation of our research interest to develop novel chalcone derivatives containing heterocycles (Yoon *et al.*, 2012) the crystal structure of the title compound was determined.

The molecular structure of the title compound is shown in Fig. 1. The fused dihydropyran ring is in a half-chair conformation with atoms O2 and C3 positioned 0.1318 (13) and 0.143 (2)Å respectively, either side of the mean plane of the other four atoms (C2/C4/C10/C11). In the crystal, weak C—H···O hydrogen bonds link molecules along [001] (Fig. 2). Examples of structures of chromene compounds have been published (Yusufzai *et al.*, 2012; Betz *et al.*, 2011; Bardajee *et al.*, 2007).

### Experimental

To a solution of 2-hydroxy-3-methoxy-benzaldehyde (1.52 g, 10 mmol) in 20 ml of 1,4-dioxane was added excess amount of acrolein (840 mg, 15 mmol) and potassium carbonate (1.4 g, 10 mmol) at room temperature. The reaction mixture was refluxed for 8 h and TLC showed no starting material of 2-hydroxy-3-methoxy-benzaldehyde. After cooling to room temperature, the mixture was poured into iced water (40 ml) and extracted with diethylether (3 × 30 ml) and combined organic layers were dried under MgSO_4_. Filtration, evaporation of filtrate gave residue which was purified by flash chromatography to give the title compound (1.21 g, 82%). Recrystallization of a solution of the title compound in ethanol gave pale yellow crystals (mp: 352-353K).

### Refinement

The H atoms were placed in calculated positions and refined as riding with C–H = 0.95 A [*U*_iso_(H) = 1.2 *U*_eq_(C)].

### Crystal data

**Table:**

C_11_H_10_O_3_	*F*(000) = 800
*M**_r_* = 190.19	*D*_x_ = 1.381 Mg m^−^^3^
Orthorhombic, *P**b**c**a*	Mo *K*α radiation, λ = 0.71073 Å
Hall symbol: -P 2ac 2ab	Cell parameters from 3190 reflections
*a* = 6.8940 (6) Å	θ = 3.1–28.2°
*b* = 13.2079 (11) Å	µ = 0.10 mm^−^^1^
*c* = 20.0964 (16) Å	*T* = 200 K
*V* = 1829.9 (3) Å^3^	Block, pale yellow
*Z* = 8	0.23 × 0.21 × 0.19 mm

### Data collection

**Table:**

Bruker SMART CCD diffractometer	1194 reflections with *I* > 2σ(*I*)
Radiation source: fine-focus sealed tube	*R*_int_ = 0.056
Graphite monochromator	θ_max_ = 28.3°, θ_min_ = 2.0°
φ and ω scans	*h* = −9→9
12690 measured reflections	*k* = −17→16
2276 independent reflections	*l* = −26→19

### Refinement

**Table:**

Refinement on *F*^2^	Primary atom site location: structure-invariant direct methods
Least-squares matrix: full	Secondary atom site location: difference Fourier map
*R*[*F*^2^ > 2σ(*F*^2^)] = 0.051	Hydrogen site location: inferred from neighbouring sites
*wR*(*F*^2^) = 0.158	H-atom parameters constrained
*S* = 0.92	*w* = 1/[σ^2^(*F*_o_^2^) + (0.0862*P*)^2^] where *P* = (*F*_o_^2^ + 2*F*_c_^2^)/3
2276 reflections	(Δ/σ)_max_ = 0.001
128 parameters	Δρ_max_ = 0.28 e Å^−^^3^
0 restraints	Δρ_min_ = −0.28 e Å^−^^3^

### Special details

**Table:**

Geometry. All e.s.d.'s (except the e.s.d. in the dihedral angle between two l.s. planes) are estimated using the full covariance matrix. The cell e.s.d.'s are taken into account individually in the estimation of e.s.d.'s in distances, angles and torsion angles; correlations between e.s.d.'s in cell parameters are only used when they are defined by crystal symmetry. An approximate (isotropic) treatment of cell e.s.d.'s is used for estimating e.s.d.'s involving l.s. planes.
Refinement. Refinement of *F*^2^ against ALL reflections. The weighted *R*-factor *wR* and goodness of fit *S* are based on *F*^2^, conventional *R*-factors *R* are based on *F*, with *F* set to zero for negative *F*^2^. The threshold expression of *F*^2^ > σ(*F*^2^) is used only for calculating *R*-factors(gt) *etc*. and is not relevant to the choice of reflections for refinement. *R*-factors based on *F*^2^ are statistically about twice as large as those based on *F*, and *R*- factors based on ALL data will be even larger.

### Fractional atomic coordinates and isotropic or equivalent isotropic displacement parameters (Å^2^)

**Table:**

	*x*	*y*	*z*	*U*_iso_*/*U*_eq_	
O1	0.1026 (2)	0.40157 (12)	0.04444 (7)	0.0583 (5)	
C1	0.1052 (3)	0.47227 (17)	0.08309 (9)	0.0456 (5)	
H1	0.1013	0.5387	0.0651	0.055*	
C2	0.1139 (2)	0.46201 (14)	0.15445 (8)	0.0351 (4)	
C3	0.1208 (3)	0.35793 (14)	0.18379 (8)	0.0381 (5)	
H3A	0.0257	0.3148	0.1601	0.046*	
H3B	0.2512	0.3291	0.1757	0.046*	
O2	0.08111 (19)	0.35301 (9)	0.25332 (6)	0.0438 (4)	
C4	0.1089 (2)	0.43707 (13)	0.29215 (8)	0.0318 (4)	
C5	0.1082 (2)	0.42242 (14)	0.36068 (9)	0.0340 (4)	
O3	0.09121 (18)	0.32461 (10)	0.38224 (6)	0.0450 (4)	
C6	0.0786 (3)	0.30827 (18)	0.45209 (9)	0.0530 (6)	
H6A	0.1980	0.3320	0.4735	0.080*	
H6B	0.0616	0.2358	0.4609	0.080*	
H6C	−0.0324	0.3458	0.4700	0.080*	
C7	0.1241 (2)	0.50626 (15)	0.40230 (9)	0.0394 (5)	
H7	0.1240	0.4974	0.4492	0.047*	
C8	0.1403 (3)	0.60283 (15)	0.37558 (9)	0.0449 (5)	
H8	0.1496	0.6597	0.4043	0.054*	
C9	0.1428 (3)	0.61667 (15)	0.30801 (9)	0.0400 (5)	
H9	0.1565	0.6829	0.2902	0.048*	
C10	0.1255 (2)	0.53375 (13)	0.26518 (8)	0.0325 (4)	
C11	0.1184 (2)	0.54390 (14)	0.19384 (9)	0.0351 (4)	
H11	0.1170	0.6095	0.1745	0.042*	

### Atomic displacement parameters (Å^2^)

**Table:**

	*U*^11^	*U*^22^	*U*^33^	*U*^12^	*U*^13^	*U*^23^
O1	0.0741 (11)	0.0674 (11)	0.0334 (8)	0.0080 (8)	−0.0032 (7)	−0.0087 (7)
C1	0.0500 (12)	0.0538 (14)	0.0330 (11)	0.0056 (9)	−0.0001 (9)	0.0023 (9)
C2	0.0338 (10)	0.0450 (12)	0.0266 (10)	−0.0007 (8)	0.0004 (7)	0.0008 (8)
C3	0.0505 (12)	0.0364 (11)	0.0274 (10)	−0.0032 (8)	0.0029 (8)	−0.0036 (7)
O2	0.0709 (10)	0.0331 (8)	0.0275 (7)	−0.0052 (6)	0.0046 (6)	−0.0021 (5)
C4	0.0335 (10)	0.0324 (10)	0.0294 (10)	0.0004 (7)	0.0009 (7)	−0.0034 (7)
C5	0.0367 (10)	0.0368 (11)	0.0285 (10)	0.0030 (8)	0.0018 (7)	0.0038 (7)
O3	0.0620 (9)	0.0394 (8)	0.0335 (8)	0.0043 (6)	0.0047 (6)	0.0069 (6)
C6	0.0680 (14)	0.0563 (14)	0.0347 (11)	0.0109 (11)	0.0086 (9)	0.0144 (9)
C7	0.0438 (11)	0.0480 (12)	0.0264 (9)	0.0043 (9)	−0.0004 (7)	−0.0029 (8)
C8	0.0536 (12)	0.0419 (12)	0.0392 (11)	−0.0009 (9)	0.0001 (9)	−0.0124 (9)
C9	0.0501 (12)	0.0333 (11)	0.0365 (10)	0.0001 (8)	0.0021 (8)	−0.0017 (8)
C10	0.0313 (9)	0.0359 (11)	0.0304 (10)	0.0011 (7)	0.0012 (7)	−0.0011 (7)
C11	0.0379 (10)	0.0359 (11)	0.0315 (10)	0.0001 (8)	0.0004 (7)	0.0058 (7)

### Geometric parameters (Å, º)

**Table:**

O1—C1	1.215 (2)	O3—C6	1.423 (2)
C1—C2	1.442 (2)	C6—H6A	0.9800
C1—H1	0.9500	C6—H6B	0.9800
C2—C11	1.341 (3)	C6—H6C	0.9800
C2—C3	1.497 (3)	C7—C8	1.388 (3)
C3—O2	1.425 (2)	C7—H7	0.9500
C3—H3A	0.9900	C8—C9	1.370 (2)
C3—H3B	0.9900	C8—H8	0.9500
O2—C4	1.370 (2)	C9—C10	1.398 (2)
C4—C5	1.391 (2)	C9—H9	0.9500
C4—C10	1.392 (2)	C10—C11	1.441 (2)
C5—O3	1.368 (2)	C11—H11	0.9500
C5—C7	1.392 (3)		
			
O1—C1—C2	124.4 (2)	O3—C6—H6B	109.5
O1—C1—H1	117.8	H6A—C6—H6B	109.5
C2—C1—H1	117.8	O3—C6—H6C	109.5
C11—C2—C1	120.83 (18)	H6A—C6—H6C	109.5
C11—C2—C3	120.50 (16)	H6B—C6—H6C	109.5
C1—C2—C3	118.66 (16)	C8—C7—C5	120.32 (17)
O2—C3—C2	114.96 (14)	C8—C7—H7	119.8
O2—C3—H3A	108.5	C5—C7—H7	119.8
C2—C3—H3A	108.5	C9—C8—C7	120.46 (18)
O2—C3—H3B	108.5	C9—C8—H8	119.8
C2—C3—H3B	108.5	C7—C8—H8	119.8
H3A—C3—H3B	107.5	C8—C9—C10	120.29 (18)
C4—O2—C3	119.63 (13)	C8—C9—H9	119.9
O2—C4—C5	116.79 (16)	C10—C9—H9	119.9
O2—C4—C10	122.21 (16)	C4—C10—C9	119.08 (17)
C5—C4—C10	120.89 (16)	C4—C10—C11	118.04 (16)
O3—C5—C4	116.44 (16)	C9—C10—C11	122.86 (17)
O3—C5—C7	124.61 (16)	C2—C11—C10	120.88 (17)
C4—C5—C7	118.95 (17)	C2—C11—H11	119.6
C5—O3—C6	117.45 (15)	C10—C11—H11	119.6
O3—C6—H6A	109.5		
			
O1—C1—C2—C11	179.25 (18)	C4—C5—C7—C8	0.1 (2)
O1—C1—C2—C3	0.2 (3)	C5—C7—C8—C9	−0.7 (3)
C11—C2—C3—O2	15.8 (2)	C7—C8—C9—C10	1.2 (3)
C1—C2—C3—O2	−165.19 (15)	O2—C4—C10—C9	176.48 (15)
C2—C3—O2—C4	−23.3 (2)	C5—C4—C10—C9	0.4 (2)
C3—O2—C4—C5	−166.65 (15)	O2—C4—C10—C11	−1.8 (2)
C3—O2—C4—C10	17.1 (2)	C5—C4—C10—C11	−177.88 (14)
O2—C4—C5—O3	3.7 (2)	C8—C9—C10—C4	−1.1 (3)
C10—C4—C5—O3	179.99 (15)	C8—C9—C10—C11	177.14 (16)
O2—C4—C5—C7	−176.19 (15)	C1—C2—C11—C10	179.49 (15)
C10—C4—C5—C7	0.1 (2)	C3—C2—C11—C10	−1.5 (2)
C4—C5—O3—C6	−176.38 (15)	C4—C10—C11—C2	−6.0 (2)
C7—C5—O3—C6	3.5 (2)	C9—C10—C11—C2	175.80 (17)
O3—C5—C7—C8	−179.85 (16)		

### Hydrogen-bond geometry (Å, º)

**Table:**

*D*—H···*A*	*D*—H	H···*A*	*D*···*A*	*D*—H···*A*
C6—H6*B*···O1^i^	0.98	2.49	3.340 (3)	145

Symmetry code: (i) *x*, −*y*+1/2, *z*+1/2.
